# The Increase in Lunacy

**Published:** 1897-07-24

**Authors:** 


### MODERN SOCIOLOGY.

### THE INCREASE IN LUNACY.

FOR many years past the steady increase in the number of insane persons coming under the care of the authorities has given rise to considerable anxiety. Not only has the growing burden thus imposed on the public purse been a matter for consideration, but in view of the figures displayed by official returns year after year it has been impossible to avoid asking the question whether our civilisation was not after all entailing upon us evil consequences such as would far more than counterbalance any benefits which we gained from it, for it seemed that however great might be the increase in wealth, the mind of the nation was giving way.

In 1859 the number of lunatics, idiots, and persons of unsound mind, reported to the lunacy department as resident in asylums and other establishments for the insane and in workhouses, or with their relatives or others, was 36,762, while in 1896 the number had increased to 96,446. Thus the amount of known insanity had nearly trebled itself in the course of less than forty years.

The question then becomes an anxious one. Is insanity occurring out of proportion to the increase of population, and so bringing with it a serious menace to the race, or is the increase apparent only and the product of the social and other changes in the modes of dealing with the insane? This latter is the question which the Commissioners in Lunacy have endeavoured to answer in a Special Report on the Alleged Increase of Insanity, lately presented to the Lord Chancellor.

The case for the decadence of the race is certainly very strong. Not only has there been the great increase in the number of known lunatics which we have already mentioned, but it has been a progressive one, the increase being of late at the rate of more than 2,000 a year. Of course, the population has grown, but that will not account for the increase in the *proportion* of the insane, for the ratio of known lunatics to population, which in 1859 was 18·67 per 10,000, had risen in 1896 to 31·38 per 10,000. Again, if we take the number of separate admissions the same tale is told, there being an increase from 4·71 per 10,000 in 1869 to 6·09 in 1895. Some of these, however, might be thought to be old cases readmitted, so the "first cases" have been picked out, with the result that they also are seen to have increased in proportion to the population from 3·3 to 4·0 per 10,000.

Now for explanations. To begin with, it is clear that there has been a steady transference of cases from the unregistered to the registered class going on all this time. The number of insane persons recorded in the census is always larger than that of the persons registered by the Commissioners in Lunacy. Yet notwithstanding that the census figures not only increase time after time, but have increased out of proportion to the growth of the population, the difference between the census lunacy and the registered lunacy has diminished both in actual numbers and in ratio, the difference in 1871 being equal to 17·7 per cent., while in 1891 it is only equal to 10·8 per cent., so that evidently there has been a very large transference from one class to the other, all of which must help to account for the increase in the proportion of registered lunacy to population independently of any actual increase in lunacy having occurred. The increase in the number of first cases cannot, however, be thus explained, but the Commissioners see ground for thinking that the explanation of this lies in the fact that while there has been a steady increase in the number of lunatics in asylums, the number of those in workhouses and residing with relatives and others has diminished equally steadily, and it seems not unlikely that when admitted to asylums a large number of these would go to swell the percentage of those stated to be suffering from "first attacks."

Another matter of importance is the "age distribution" of the insane compared with that of the rest of the population. The time of life at which there is the greatest liability to attacks of insanity is from twenty to forty-five. Now, between 1881 and 1891 a change took place in the age distribution of the population, so that at the latter date the number of people living at these ages was larger in proportion to the whole mass of the population than it had been ten years before. But it was otherwise with the lunatics; not only was the proportion of the insane at these ages to the whole number of the insane less than it had been ten years before, but the ratio of the insane at various ages to the total population at the same ages is shown to have been less in 1891 than in 1881 for all ages below forty-five, and markedly greater for all ages above forty-five. This diminution of the ratio of the insane to the rest of the population at those ages which are most liable to the occurrence of insanity, coupled with the increase of the ratio at later ages, is strong evidence that we are having to do with an accumulation of old cases rather than with an increased production of new ones.

The causes of the apparent increase of lunacy may then be stated to be due to the (1) greater accuracy of registration; (2) extended views as to what constitutes insanity requiring confinement; (3) the retention in workhouses of a diminished proportion of pauper lunatics; (4) the four shillings grant, "a relief of local taxation, which in our opinion has largely contributed by its reactions to increase the burden of lunacy upon the public"; (5) the increasing popularity of asylums; (6) the increasing ratio of transfers from the unregistered to the registered class, with consequent reduction in the number of insane persons cared for at home; (7) the increasing proportion admitted of old and broken down cases; (8) the density of population in towns and the decrease in home industries, making it less possible to retain the slighter cases of insanity at home; (9) the increased use of asylums for the treatment of temporary attacks of alcoholic insanity; of this there is ample evidence; (10) the opening of new asylums; (11) diminution in the number discharged, either as uncured or as cured, and also in the death-rate.

The Commissioners come to the conclusion that these considerations are sufficient to explain the apparent increase of insanity, and they state that they are unable to satisfy themselves that there has been any important increase of occurring or fresh cases. It is well known that this has long been the official view, but it is satisfactory to have the whole grounds of the belief so clearly set out as is done in this report.

